# *Helicobacter pylori* components increase the severity of metabolic syndrome and its hepatic manifestations induced by a high fat diet

**DOI:** 10.1038/s41598-024-56308-7

**Published:** 2024-03-08

**Authors:** Agata Tomaszewska, Weronika Gonciarz, Tomasz Rechcinski, Magdalena Chmiela, Anna K. Kurdowska, Agnieszka Krupa

**Affiliations:** 1 Department of Immunology and Infectious Biology, Faculty of Biology and Environmental Protection, University of Lodz, Lodz, Poland; 2 Bio-Med-Chem Doctoral School of the University of Lodz and Lodz Institutes of the Polish Academy of Sciences, University of Lodz, Lodz, Poland; 3 1st Department of Cardiology, Medical University of Lodz, Lodz, Poland; 4 Department of Cellular and Molecular Biology, The University of Texas Health Science Center at Tyler, Tyler, Texas USA

**Keywords:** Atherosclerosis, Metabolic syndrome, *Helicobacter pylori*, Endothelium, Immunology, Microbiology, Cardiology, Gastroenterology

## Abstract

The metabolic syndrome, often accompanied by hepatic manifestations, is a high-risk factor for developing cardiovascular disease. Patients with metabolic dysfunction associated with steatohepatic disease (MASDL) are at significant risk of developing coronary artery disease. Atherosclerosis is a systemic inflammatory disorder in which several factors, including dietary or infectious factors, can cause an inflammatory response. *Helicobacter pylori* (*HP*) bacteria have been implicated in the progression of proatherogenic vascular endothelial lesions, moreover, our previous study in an experimental in vivo model of *Cavia porcellus* showed that *HP* components and high-fat substances acted synergistically in promoting vascular endothelial inflammation, leading to an early onset of a proatherogenic environment. In the present study, our goal was to determine the contribution of *HP* components to the development of hepatic manifestations of metabolic syndrome in an experimental model. Our results showed that *HP* infection in animals exposed to a high-fat diet increased oxidative stress and lipid peroxidation, followed by endothelial lipid deposition, impaired endothelial apoptosis, cell lysis, and increased vascular stiffness. Finally, histopathological analysis of liver tissue showed signs of MASLD development in *HP*-infected animals fed a high-fat diet.

## Introduction

Cardiovascular diseases (CVD) are a major cause of death in Europe. The European Heart Network reports that the number of CVD cases is still on the rise^1^. CVD includes various vascular disorders, but the most common and severe of them is atherosclerosis. This condition can develop quickly and may lead to a heart attack or stroke^1^. Atherosclerosis is a medical condition that affects the entire body and causes inflammation. It is strongly connected to high levels of oxidized low-density lipoprotein (ox-LDL). This type of LDL is involved in the development of atherosclerotic plaques in medium and large arteries, which can lead to serious health problems^2^. Multiple factors can cause the inflammatory response in atherosclerosis, including dietary or infectious sources. There is substantial evidence that the gut microbiota, which includes Escherichia *coli* (*EC*) and its lipopolysaccharide, can impact the development of atherosclerosis through Toll-like receptor 4 (TLR-4)—induced cellular oxidative stress^3–5^. In addition, research conducted through seroepidemiological data and both in vitro and in vivo studies indicate that the presence of *Helicobacter pylori* bacteria (*H. pylori*, *HP*) may impact the development of atherosclerosis. This is particularly significant considering that over half of the world's population is infected with *HP*, and the infection is common in patients with cardiovascular disease^6–13^.

Earlier studies have shown^14^, that soluble components of *H. pylori* bacteria, when combined with a high-fat diet, can create a proatherogenic environment in *Cavia porcellus* (guinea pig) that have been infected with the bacteria orally. This proatherogenic environment refers to a scenario, where the *HP* bacteria colonize the stomach, causing chronic inflammation and high levels of reactive oxygen species (ROS) to be released from activated immune cells or gastric epithelial cells that are under stress^15^. This inflammation weakens the epithelial barrier, allowing bacterial components to enter the bloodstream. Furthermore, the proatherogenic environment is characterized by the presence of oxidized lipids that undergo peroxidation in the presence of oxidative stress. This can increase inflammation and damage vascular endothelial cells, which can ultimately transform macrophages into foam cells that participate in the formation of atherosclerotic plaque as showed previously^14^. Moreover, our previous research, that was done using fluorescently labeled dextran particles, showed that the secretion of reactive oxygen species (ROS) in response to *HP* components resulted in increased permeability of human gastric epithelial cells (AGS) or human vascular endothelial cells (HUVEC) in vitro. We also found that *HP* components inhibited the regeneration of gastric epithelial cells in vitro*,* thus promoted chronic inflammation^8,15,16^. It's important to note that an imbalance between excessive ROS release and the number of neutralizing antioxidants could lead to inflammation and the development of atherosclerosis^17^. Pathogens and their soluble components can contribute to the development of atherosclerosis by not only inducing inflammation but also affecting lipid metabolism. This can be observed through an increase in LDL or triglyceride levels and a decrease in the concentration of high-density lipoproteins (HDL)^4^. Research has shown that patients with diagnosed cardiovascular disease who are infected with *HP* have significantly increased cholesterol levels, which decrease after the eradication of these pathogenic bacteria^18^. *HP* incorporates non-esterified cholesterol into the cell membrane and induces its glycosylation, using both non-esterified and glycosylated cholesterol as membrane lipid composition. This enables *HP* to evade phagocytosis and detoxify sterols that could be fatal to the bacterium^19^. In the stomach, *HP* reduces the amount of cholesterol in the gastric glands. This depletion helps to prevent interferon gamma signaling and allows the bacteria to escape the inflammatory response of the host^20^. Additionally, some studies have suggested that atherosclerosis may be accompanied by an autoimmune response to low-density lipoprotein (LDL) or various antigens of infectious agents, including *HP*. This response may lead to the induction of cross-reactive antibodies^10,21,22^. When the concentration of LDL (low-density lipoprotein) in the bloodstream increases, there is a rise in the uptake of this lipid fraction by the cells, leading to the accumulation of cholesterol in the endothelium^23,24^. This, in turn, results in the deposition of cholesterol in the vessels, primarily through interactions with proteoglycans of the subendothelial matrix. Oxidized LDL is classified as a damage-associated molecular pattern (DAMP), and its recognition by lectin-like oxidized low-density lipoprotein receptor-1 (LOX-1) and other pattern recognition receptors (PRRs) can activate endothelial cells. This activation causes an upregulation of cytokines and chemokines that initiate the recruitment of monocytes^25^. These monocytes, upon taking up oxidized LDL, transform into foam cells, which further upregulate endothelial inflammation^17,24,26^. In addition to cholesterol, lipid oxidation can produce other harmful substances like 7-ketocholesterol (7KCh) and aldehydes such as 4-hydroxy-2-nonenal (4-HNE), which have proatherogenic effects. These substances can react with proteins and activate signaling pathways that damage cell membranes ^27^. In a previous study, we found that *HP* components can cause macrophages to turn into foam cells in the presence of oxidized cholesterol-7KCh^14^. Another study showed that 7KCh can decrease the integrity of the HUVEC monolayer and increase cell apoptosis^8^. Recent studies indicate that metabolic syndrome, which is often accompanied by CVD, can also manifest in the liver, resulting in a high risk of clinical symptoms of CVD^28,29^. Growing evidence suggests that patients with Metabolic Dysfunction-Associated Steatotic Liver Disease (MASLD), previously known as Non-Alcoholic Fatty Liver Disease (NAFLD), are at significant risk of developing coronary heart disease, cardiomyopathy, and cardiac arrhythmias. This can ultimately lead to increased cardiovascular morbidity and mortality^28,30–32^. The inflammatory aspect of atherosclerosis plays a crucial role in the development of cardiovascular disease. The hepatic manifestation of the metabolic syndrome is becoming increasingly common in patients with CVD. Therefore, the question of how inflammation is initiated and how it contributes to the development of the set of biochemical and physiological abnormalities associated with CVD is of great interest. The role *HP* infection in driving these events is also being investigated. Several animal models, including the guinea pig model, are being used to study the role of various factors, such as increased fat intake or infectious agents, in the development of CVD^33^. We conducted a study to investigate the impact of *HP* infection induced locally in the stomach of guinea pigs fed a high-fat diet. We aimed to determine whether this could lead to increased oxidative stress and lipid peroxidation, followed by lipid deposition in the endothelium. We also wanted to examine whether this process could result in a range of functional impairments, such as deregulation of apoptosis, endothelial breakdown, and increased vascular stiffness. Additionally, we sought to explore the potential effects of increased deposition of oxidized lipids on the surface expression of intracellular adhesion molecule-1 (ICAM-1), which is responsible for allowing inflammatory cells to interact with the vascular endothelium or may be involved in regenerative processes.

Finally, we also aimed to investigate whether vascular endothelial events that are dependent on increased fat intake and ongoing *HP* infection are reflected in hepatic symptoms. These symptoms have been suggested as early markers of CVD.

## Results

To study the relationship between inflammation caused by *HP* bacteria and the development of metabolic syndrome, a group of *Cavia porcellus* animals was used in an in vivo model where they were either infected with *HP* or left uninfected. They were then fed either a high-fat or standard diet. Helicobacter-like organisms (HLO) were found in gastric tissue of all *HP*-infected animals, regardless of the type of diet, 7, 28, or 60 days after inoculation. The *HP*-infected animals developed gastric inflammation, which was primarily caused by granulocytes and lymphocytes in the later phases of infection. Specific anti-*HP* antibodies were produced by the animals and were detectable in their serum samples at 7, 28, and 60 days after inoculation. Some animals were observed for up to 120 days to track the late systemic effects of *HP* infection in the context of the development of metabolic syndrome, but no further investigation of *HP* infection markers was conducted.

### Systemic inflammatory response in *Cavia porcellus* in response to oral inoculation with *Helicobacter pylori* bacilli

**Figure 1:**
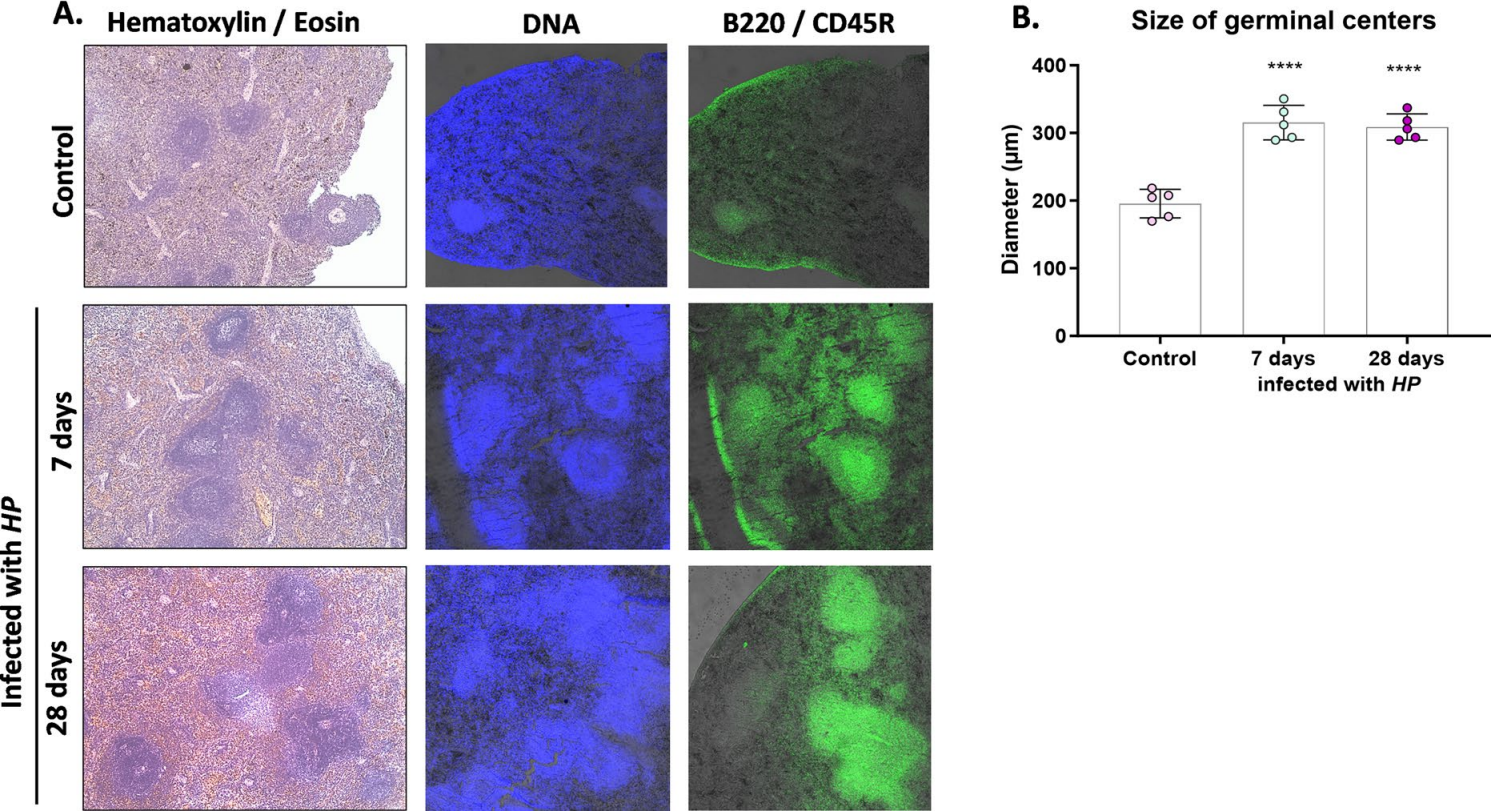
Size of germinal centers in the spleens of animals at 7 and 28 days after *H. pylori* (*HP*) infection. (**A**) Representative images of germinal centers in the spleen of animals; B220/cluster differentiation CD45R (excitation 495 nm, emission 519 nm; green)—a marker of B lymphocyte differentiation; DAPI (excitation 358 nm, emission 461 nm; blue)—staining of nuclei. (**B**) Diameter of germinal centers in the animals' spleens; Statistical analysis was performed using one-way ANOVA, and Dunnett's post-hoc multiple comparisons test ****p < 0.0001; Comparison: control animals (not infected) vs guinea pigs at 7 and 28 days after *HP* inoculation.

We conducted a study to assess whether exposure of animals to *HP* infection resulted in increased activation of systemic immune cells. To do this, we examined the germinal centers of the animals' spleens either 7 or 28 days following *HP* inoculation or in uninfected animals. We found that the size of germinal centers, as measured by mean diameter, increased during *HP* infection, and this difference was statistically significant at both 7 and 28 days after *HP* inoculation in guinea pigs (Fig. 1A, B). Additionally, the number of germinal centers also increased during *HP* infection. In control animals, the average number of germinal centers per 4 mm2 was less than 10, whereas 28 days after *HP* inoculation it was more than 11 (data not shown).

### Low-density lipoprotein level in serum samples of *Cavia porcellus* infected with *HP* and exposed to a high-fat diet or standard chow

**Figure 2:**
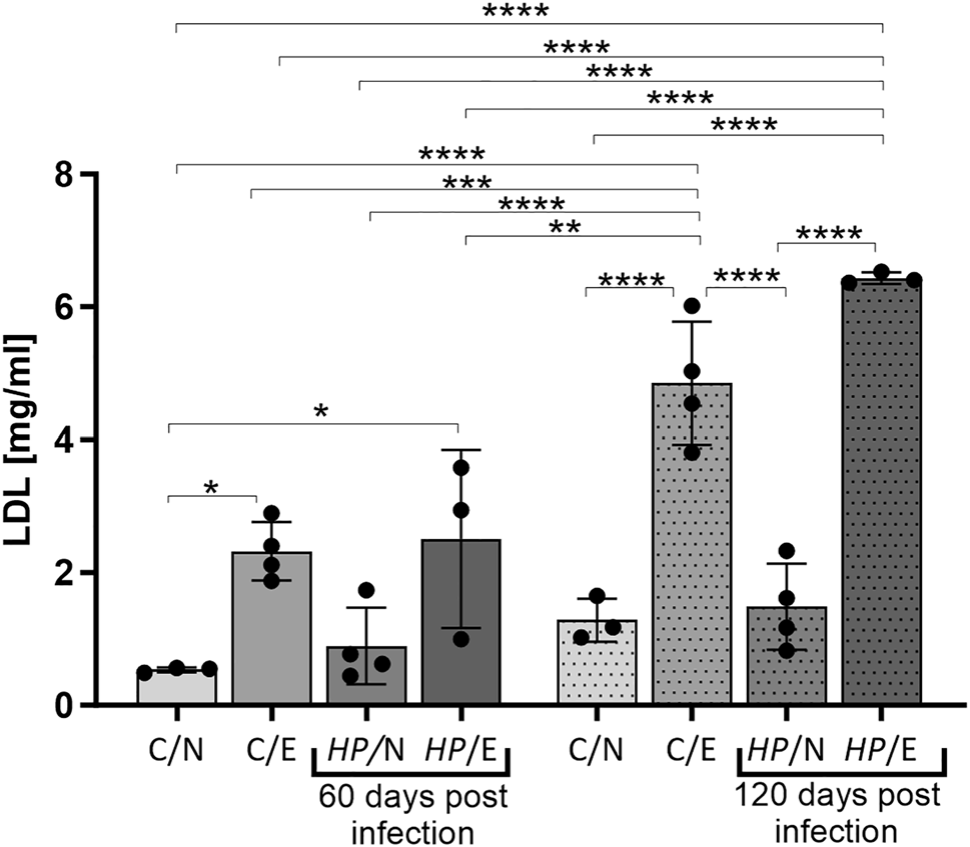
LDL concentration in serum samples of animals: uninfected (C-control) with *H. pylori* (*HP*) on a normal (N) diet (C/N), uninfected (C-control) on an experimental (E) high-fat diet (C/E), infected with *HP* on a normal diet (*HP*/N), infected with *HP* on an experimental high-fat diet (*HP*/E). Results are presented as the mean values ± SD. Statistical analysis was performed with three-way ANOVA followed by post-hoc Tukey's multiple comparisons test. *p = 0.0332; **p = 0.0021 ***p = 0.0002; ****p < 0.0001.

We conducted a study to examine the impact of inflammation resulting from *HP* infection on metabolic syndrome related to lipids. To do this, we measured the levels of LDL in the serum of tested animals and the deposition of oxidized LDL in their aortic tissue. After 60 or 120 days of feeding, we found that the levels of LDL in the serum of animals that were exposed to a high-fat diet were significantly higher than those of animals that were fed a normal chow. This increase was observed to be time dependent. Furthermore, we found that the increase in serum LDL levels was consistently higher in animals that were fed a fat-enriched chow and infected with *HP*, as shown in Fig. 2.

### Oxidative stress and deposition of oxidized lipids in the aortic tissue of *Cavia porcellus* infected with *HP* and exposed to a high-fat diet or standard feed

**Figure 3:**
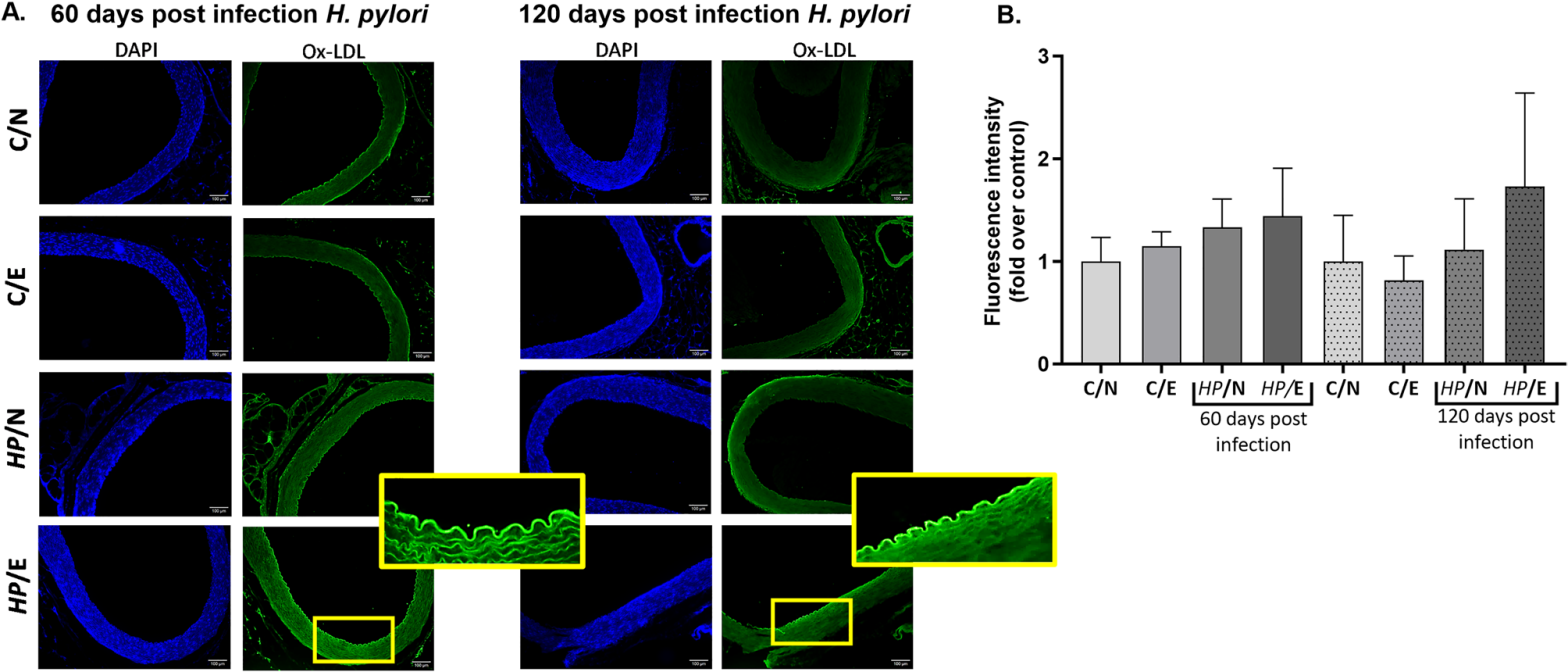
Deposition of ox-LDL in the aortic tissue of the studied animals after 60 or 120 days of the experiment. (**A**) Representative images of aortic tissue of animals: uninfected (C-control) with *H. pylori* (*HP*) on a normal (N) diet (C/N), uninfected (C-control) on an experimental (E) high-fat diet (C/E), infected with *HP* on a normal diet (*HP*/N), infected with *HP* on a high-fat diet (*HP*/E). Oxidized low density lipoprotein (ox-LDL)—green (excitation 495 nm, emission 519 nm), DAPI—(excitation 358 nm, emission 461 nm) nuclei staining, blue. (**B**) Relative fluorescence units presented as a multiple relative to the control group of animals (C/N). Results are presented as the mean values ± SD. Statistical analysis was performed on log transformed data to attain heteroscedasticity of residuals, with three-way ANOVA followed by posthoc Tukey`s multiple comparisons test.

The deposition of ox-LDL in aortic tissue was examined using specific anti-ox-LDL antibodies and immunofluorescence staining under a microscope. After 120 days of the experiment, ox-LDL deposition was visible in the aortic endothelial layer of *HP*/E animals (Fig. 3A, B), which correlated with the concentration of LDL in serum, which was highest in the *HP*/E group of animals (Fig. 2). However, the measurement of fluorescence intensity in areas where ox-LDL deposition was confirmed microscopically did not show any statistically significant difference between experimental groups, probably due to lower sensitivity resulting from the method of performing the measurement itself (Fig. 3B).

**Figure 4:**
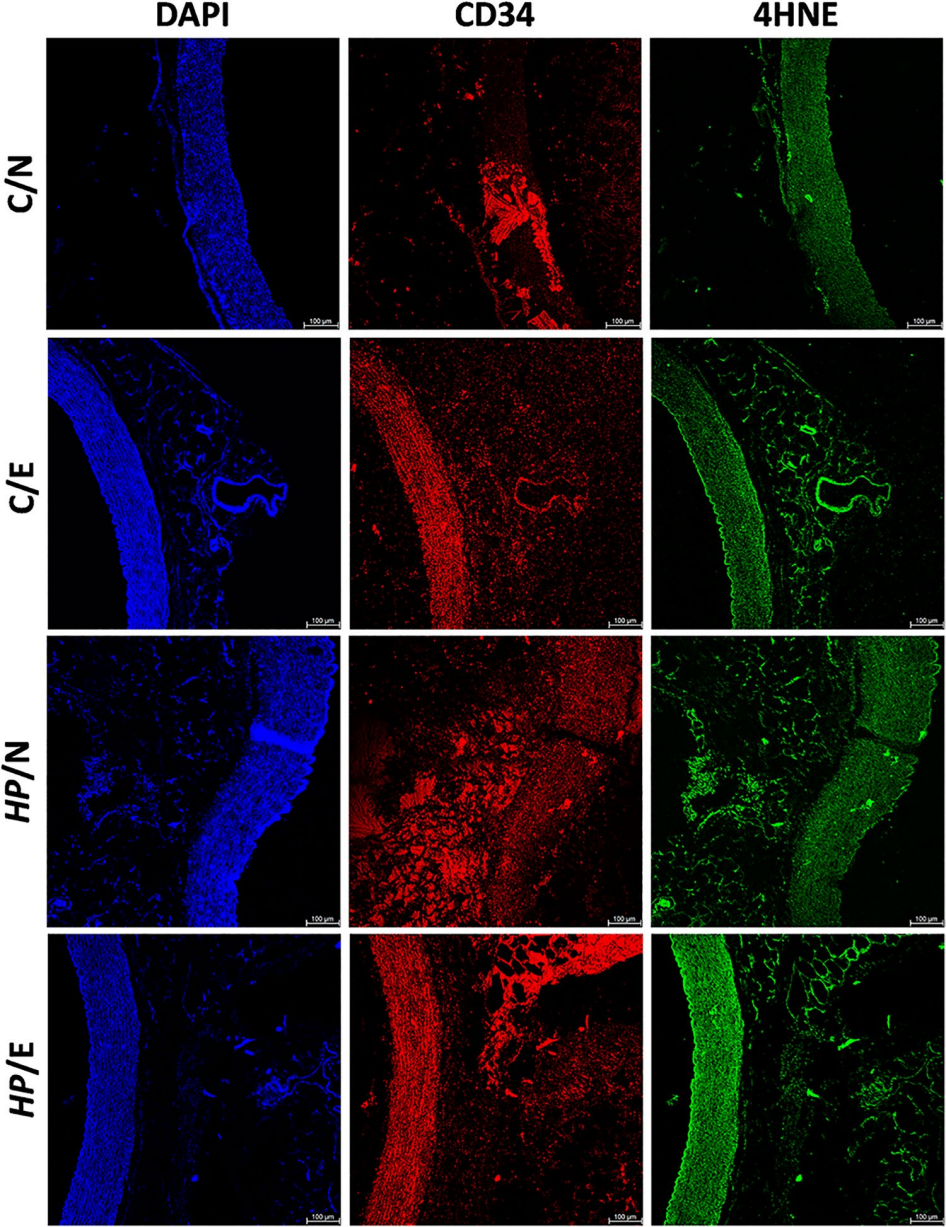
Representative images of guinea pig aortic tissue stained for 4 hydroxy 2 nonenal (4HNE). Animals: uninfected (C-control) with *H. pylori* (*HP*) on a normal (N) diet (C/N), uninfected (C-control) on experimental (E) high-fat die (C/E), infected with *HP* on a normal diet (*HP*/N), infected with *HP* on an experimental high-fat diet (*HP*/E). 4 hydroxy 2 nonenal (4HNE)—(excitation 495 nm, emission 519 nm), green; cluster of differentiation CD34—(excitation 590 nm, emission 618 nm) marker of endothelial cells, red. DAPI, marker of cell nuclei, blue (excitation 358 nm, emission 461 nm).

We experimented to evaluate the level of 4 hydroxy 2 nonenal (4HNE), a product of fatty acid peroxidation, in the aortic tissue of animals from different study groups using immunofluorescence staining. After staining aortic tissue with specific fluorescently labeled anti-4HNE antibodies, the fluorescence intensity of 4HNE was found to be highest in the group of animals infected with *HP* and fed high-fat diets (*HP*/E), as shown in Fig. 4. The images presented were taken after 60 days of feeding and/or inoculation of *HP* animals. Although CD34 was used as an endothelial cell marker, it was difficult to attribute the fluorescence signal specifically to endothelial cells as the process of lipid peroxidation may also affect smooth muscle cells^34^. These findings further support our previous observations^14^, which demonstrated that a combination of dietary (high-fat foods) and bacterial (soluble *HP* antigens) components promotes a proatherogenic environment including the transformation of macrophages into foam cells.

### Apoptosis levels in the aortic tissue of *Cavia porcellus *infected with *HP* and exposed to a high-fat diet or standard feed

**Figure 5:**
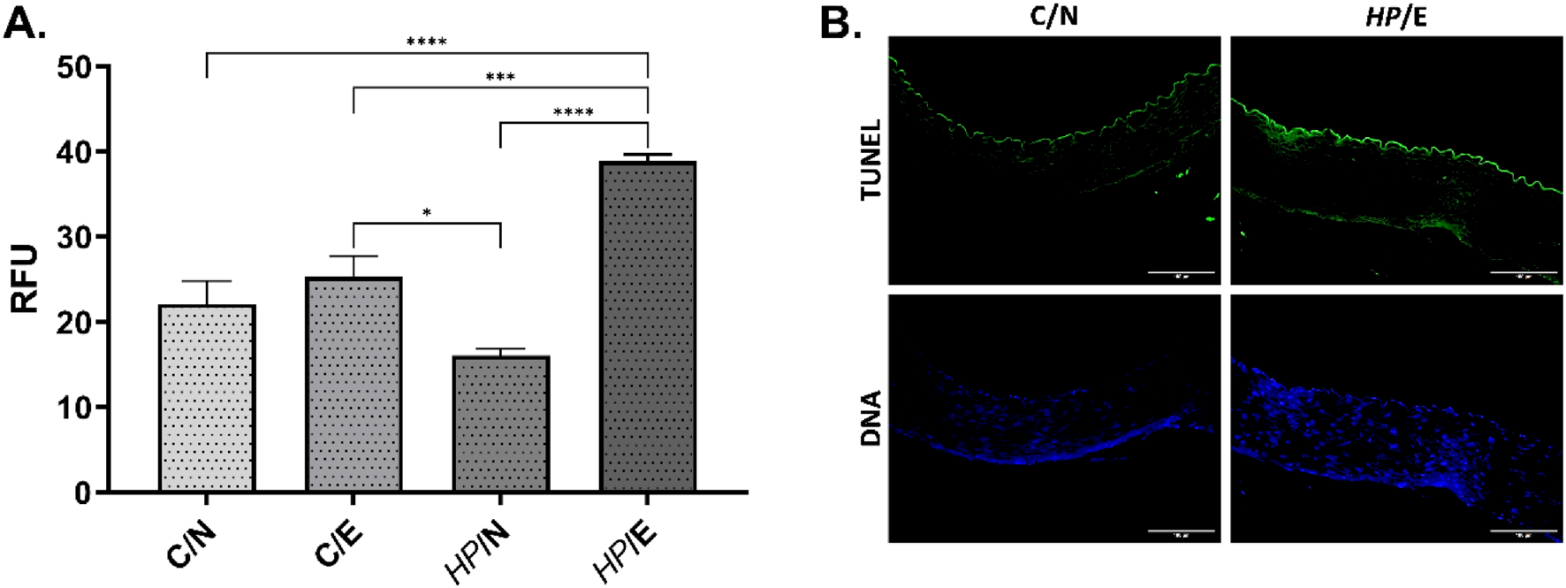
The level of apoptosis in vascular endothelium. Tested animals: uninfected (C-control) with *H. pylori* (*HP*) on a normal (N) diet (C/N), uninfected (C-control) on experimental (E) high-fat die (C/E), infected with *HP* on a normal diet (*HP*/N), infected with *HP* on an experimental high-fat diet (*HP*/E). (**A**) The intensity of fluorescence in the enzyme terminal deoxynucleotide transferase dUTP nick labelling (TUNEL) assay was calculated based on representative pictures shown as relative fluorescence units (RFU). (**B**) Representative images of aortic tissue of C/N and *HP/*E animals—TUNEL test (excitation 590 nm, emission 618 nm) endothelial apoptosis (green), DAPI—(excitation 358 nm, emission 461 nm) nucleus staining (blue). Results are presented as the mean values ± SD. Statistical analysis was performed with two-way ANOVA followed by post-hoc Tukey's multiple comparisons test. *p = 0.0332; ***p = 0.0002; ****p < 0.0001.

The study examined aortic tissues from different animal groups for apoptosis using the enzyme terminal deoxynucleotide transferase dUTP nick labelling (TUNEL) assay. The intensity of apoptosis was calculated from representative images using the Image J software. However, quantification was limited to the endothelial layer of aortic tissue. The results depicted in Fig. 5 indicate a significant increase in apoptosis in the vascular endothelium of *HP* uninfected (control) animals fed a high-fat diet (C/E group). Furthermore, it was observed that *HP*-infected guinea pigs (*HP*/E group) that were given experimental food had a much higher level of apoptosis.

### Vascular stiffness

**Figure 6:**
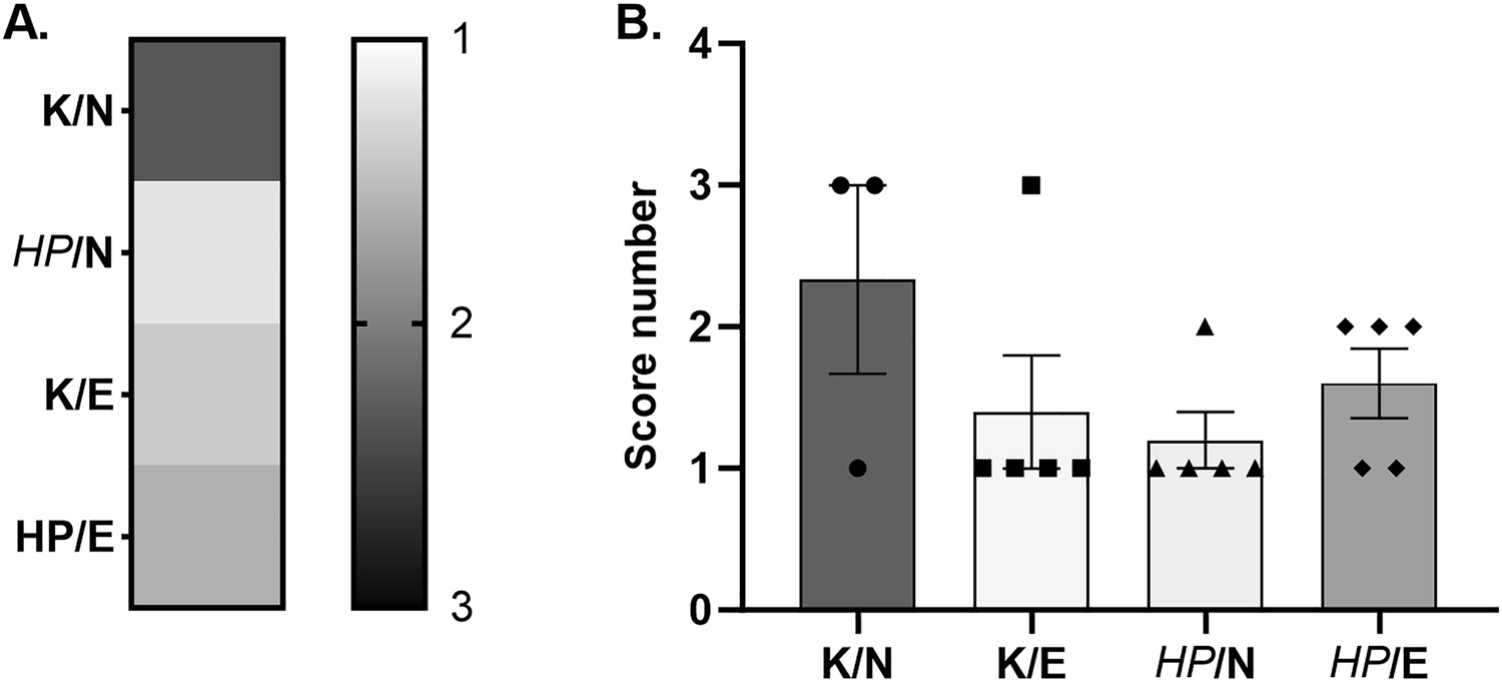
Effect of high-fat diet and/or *HP* infection on arterial stiffness in *Cavia porcellus* studied. Animals: uninfected (C-control) with *H. pylori* (*HP*) on a normal (N) diet (C/N), uninfected (C-control) on a high-fat experimental (E) diet (C/E), infected with *HP* on a normal diet (*HP*/N), infected with *HP* on a high-fat experimental diet (*HP*/E) after 120 days of feeding and/or *HP* infection. (**A**) Results shown as heat map. (**B**) Results for individual animals in different groups are shown as a bar graph (score number). Results are presented as the mean values ± SEM.

The occurrence of atherosclerosis in humans leads to an increase in blood vessel stiffness. To evaluate blood vessel stiffness in animals from different groups, we conducted a study where they were exposed to normal or fat-enriched experimental food and/or *HP* infection for 120 days. To measure the stiffness of the arteries, we used a special rodent surgical monitor with a high-resolution electrocardiogram (ECG) from Indus Animalab in Poznan, Poland^14^. The device measured the amplitude of the pulse wave, which determined the stiffness of the arteries. The lower the amplitude, the stiffer the vessel. We selected only the average amplitude value for each animal representing the data. We compared the average amplitudes between the groups and scored them based on the differences. The scoring system ranged from 1 to 3, where 1 indicates significant stiffness (amplitudes lower than 1.5 AU), 2 indicates partial arterial stiffness (amplitudes between 1.5 and 2 AU), and 3 indicates normal arterial stiffness (amplitudes between 2 and 3 AU). The results in Fig. 6 show the highest arterial stiffness (score 1) in *HP*-infected animals fed a normal diet (*HP*/N) or uninfected (control) animals that were fed a high-fat experimental diet (C/E).

### Hepatic steatosis as a hepatic manifestation of metabolic syndrome in *Cavia porcellus* infected with *HP* and exposed to a high-fat diet

Metabolic syndrome refers to a collection of biochemical and physiological abnormalities related to cardiovascular disease, that are considered to be the primary indicators of the development of atherosclerosis^35^. The most common manifestation of dysregulation of lipid metabolism in metabolic syndrome is hepatic steatosis, fibrosis, or inflammation of the liver.

**Figure 7:**
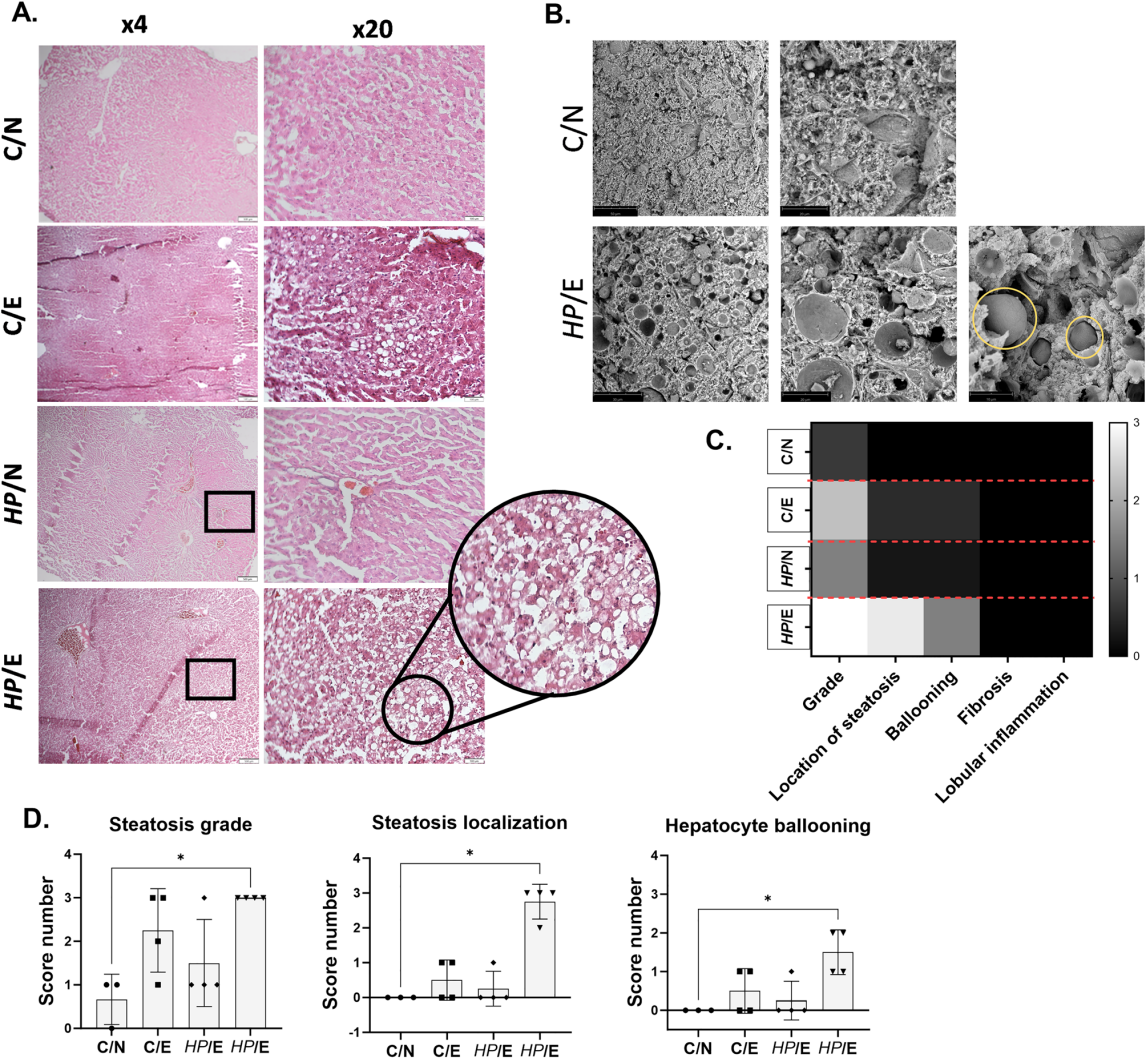
Evaluation of hepatic steatosis in animals fed a normal or fat-enriched diet and/or inoculated with *HP.* Animals: uninfected (C-control) with *H. pylori* (*HP*) on a normal (N) diet (C/N), uninfected (C-control) on a high-fat experimental (E) diet (C/E), infected with *HP* on a normal diet (*HP*/N), infected with *HP* on a high-fat experimental diet (*HP*/E) after 120 days of exposure. (**A**) Representative images of haematoxylin and eosin staining of liver sections. (**B**) Representative images of lipid droplets and steatosis in liver tissue captured using a scanning electron microscope for the C/N and *HP*/E animals' groups. (**C**) Heat map of results calculated for different symptom categories (mean values). (**D**) Results for different groups of individual animals are presented as bar graphs. Statistical analysis was performed using Kruskal–Wallis non-parametric test followed by Dunn's post-hoc multiple comparisons test. *p < 0.334.

The liver tissue of animals from different experimental groups was analyzed histopathologically after 120 days of exposure to normal or fat-enriched feed and/or *HP* (Fig. 7A)*.* The analysis was based on five parameters: degree and location of steatosis, presence of fibrosis, lobular inflammation, and presence of balloon cells (Fig. 7C, D). A score was developed based on these parameters, following the method previously described by Kleiner et al.^36^. The liver sections of animals infected with *HP* and exposed to a high-fat experimental diet (*HP*/E) showed the most intense steatosis with the highest score, location of steatosis, and ballooning. The large lipid droplets covered more than 60% of the organ surface, and lipids were mainly localized around small vessels. Ballooned hepatocytes were also present. In liver sections from C/E animals, hepatic steatosis was present as small droplets that covered less than 50% of the organ surface, with only a few balloon-shaped hepatocytes visible. There was no evidence of fibrosis or lobular inflammation in any animal, regardless of the experimental group. To visualize areas of liver steatosis in *HP-*infected animals fed a high-fat diet, we performed a scanning electron microscopy (SEM). As shown in Fig. 7B fragments of liver tissue show lipid droplets or lipid deposits in areas of parenchymal cells. In the process of steatosis, most cells accumulate lipid droplets, leading to hepatocyte ballooning and inflammation. In the process of steatosis, most parenchymal cells contain only one large fat droplet, and its volume can be many times larger than the original cell. Large fat droplets marginalize the cell's cytoplasm and organelles, which can interfere with metabolism and transport processes within the cell, and ultimately lead to the occurrence of cell necrosis. It can also be one of the methods to stop fat accumulation in cells^37^.

### Evaluation of arterial ICAM-1 expression and serum soluble ICAM-1 levels in *Cavia porcellus* fed standard or fat-enriched diets, infected or uninfected with *HP*

**Figure 8:**
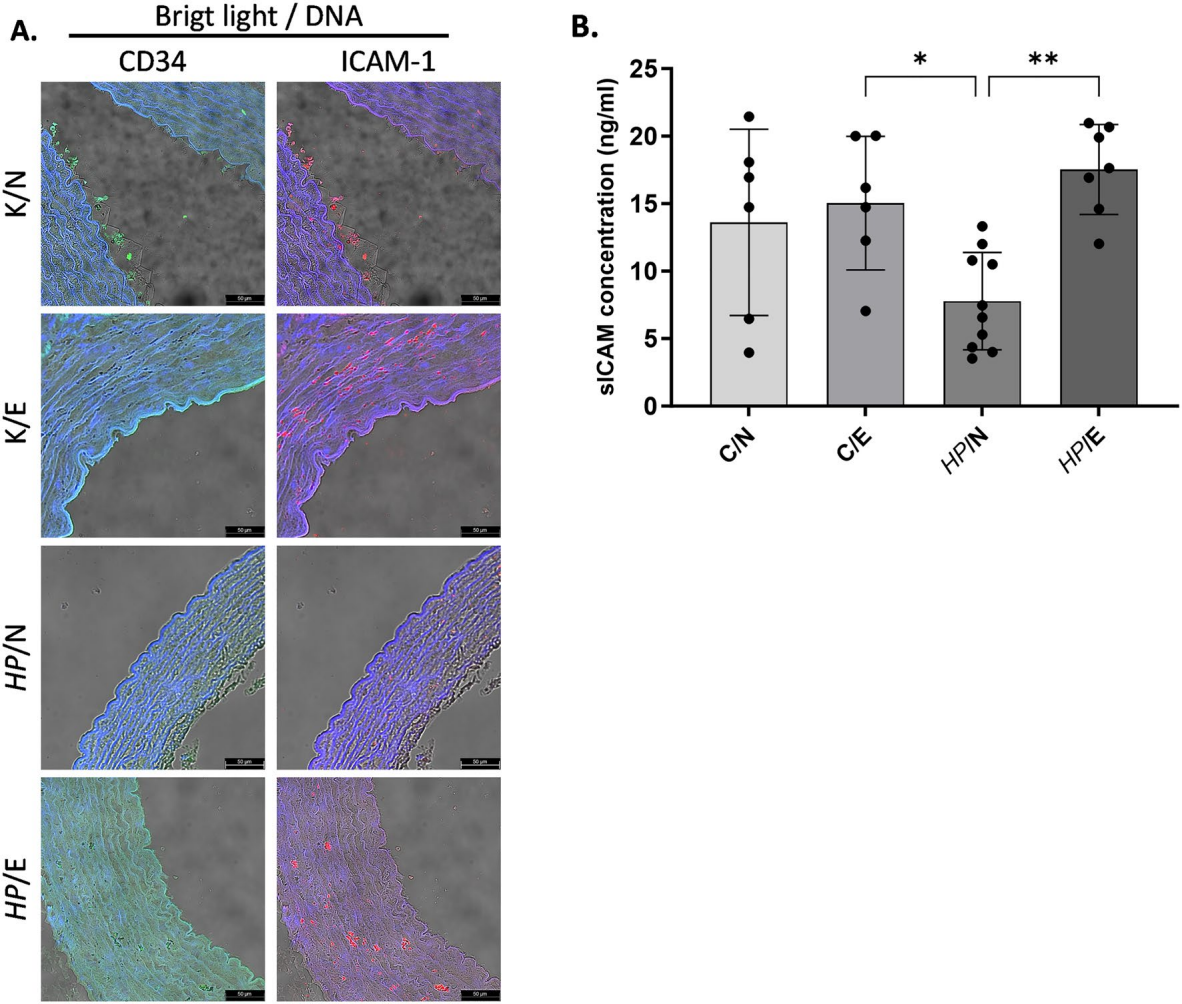
Evaluation of the expression of ICAM-1 molecule: surface or soluble in animals fed a normal or fat-enriched experimental diet and/or inoculated with *HP.* Animals: uninfected (C-control) with *HP* (*H. pylori*) on a normal (N) diet (C/N), uninfected (C-control) on a high-fat experimental (E) diet (C/E), infected with *HP* on a normal diet (*HP/*N), infected with *HP* on a high-fat experimental diet (*HP*/E) after 60 days of exposure. (**A**) Representative pictures of intracellular adhesive molecule -1 (ICAM-1) expression in aortic tissues. (**B**) Concentration of soluble ICAM-1 (sICAM-1) in animals' serum. Results are presented as the mean values ± SD. Statistical analysis was performed with two-way ANOVA followed by post-hoc Tukey's multiple comparisons test. *p = 0.0332, **p = 0.0021; ICAM-1-(excitation 590 nm, emission 618 nm) red; cluster of differentiation CD34—(excitation 495 nm, emission 519 nm) marker of endothelial cells, green.

In this study, we evaluated the expression of endothelial ICAM-1 in the aortic tissue of animals through immunofluorescence, or by measuring the level of soluble ICAM-1 in their serum using enzyme linked immunosorbent assay (ELISA). Surprisingly, feeding uninfected animals with a fat-enriched diet did not have any impact on the natural level of ICAM-1 expression in their arteries, which was like animals receiving a standard diet (Fig. 8A). However, the fluorescence of arterial samples stained with anti-ICAM-1 antibody was found to be reduced in *HP*-inoculated animals. The reduction in ICAM-1 deposition in the aortic tissue of *HP*-infected animals and those fed a fat-enriched diet was less significant than that observed in samples obtained from animals maintained on a normal diet (Fig. 8A). We used the level of soluble ICAM-1 (sICAM-1) detected in serum samples from uninfected animals fed a standard diet as a control (Fig. 8B).

The amount of sICAM-1 in the serum samples of animals fed with fat-enriched food, both infected and non-infected with *HP*, was similar to that of the control group. However, it is worth noting that the level of sICAM-1 in serum samples of *HP*-infected animals that were fed with standard feed was the lowest compared to the other experimental groups (as shown in Fig. 8B).

## Discussion

Atherosclerosis is a disease that affects the entire body and is caused by inflammation. It can be caused by various factors, including genetics, diet, and lifestyle. However, the role of microbial factors in inducing chronic inflammation is extremely important and cannot be ignored^4,7,38^. Studies show that *HP* infection is very common in patients with CVD and is associated with increased levels of anti-*H. pylori* IgG and anti-*H. pylori* IgA, which indicates chronic exposure to these bacteria^9–11^.

In this study, we found that *Cavia porcellus* infected with *HP* showed an increase in the number of splenic germinal centers. This suggests that a specific humoral response to *HP* antigens develops in infected animals. In *HP*-infected CVD patients, the humoral response is even higher than in non-CVD patients with *HP*-associated gastritis. This may indicate that the maturation and increasing number of germinal centers during *HP* infection is linked to an increased humoral response^10,39^.

Soluble components of *Helicobacter pylori* (*HP*), such as lipopolysaccharide (LPS), have been found to induce oxidative stress in vascular endothelial cells. This can lead to increased cell apoptosis, cell lysis, or proatherogenic inflammation^6,8,12^. According to Krupa et al. (2021), *HP* antigens and 7KCh antigens promote endothelial cell apoptosis through an extracellular signal-regulated kinase (ERK)-dependent signaling pathway^14^. The inflammatory response associated with *HP* infection provides an ideal environment for lipid oxidation, which drives atherogenesis. Our previous results showed that *HP* and dietary components acted synergistically in the development of an early proatherogenic endothelial milieu^14^. In an experimental model of *Cavia porcellus*, chronic *HP* infection and exposure to a high-fat experimental diet promoted the transformation of macrophages into foam cells^14^. Studies have shown that during *HP* infection, the levels of cholesterol in the body increase. However, after eradicating the infection, the cholesterol levels return to normal state. *HP* can absorb cholesterol into the cell membrane or modify cholesterol in gastric tissue to avoid an inflammatory response^18^.

Currently, to diagnose patients with a higher risk of atherosclerosis, metabolic syndrome is diagnosed in patients with obesity, hypertension, elevated triglycerides, cholesterol, low level of high density lipoprotein (HDL), high fasting glucose, or type 2 diabetes^40^. It has been suggested that patients with atherosclerosis and high LDL levels may be related to metabolic syndrome^40,41^.

In our study, we observed that serum LDL levels in animals exposed to the high-fat diet were significantly higher than in animals fed a normal diet, and the increase in LDL levels was time-dependent (higher after 120 days). Moreover, we found that the increase in serum LDL levels was consistently higher in animals fed a fat-enriched diet and infected with *HP* after 120 days (Fig. 2). Interestingly, *HP* infection alone did not significantly increase LDL levels; thus, diet was the key factor affecting serum LDL levels in the animals studied.

A study conducted by Haeri and his colleagues in 2018 revealed that the lipid profile in individuals infected with *HP* can change during infection. However, the study found that only LDL levels significantly increased in men^42^. Interestingly, a mouse model showed a direct correlation between *HP* infection and elevated LDL levels^43^, which is consistent with the results obtained in the *Cavia porcellus* model. The data indicated that animals exposed to a fat-enriched diet and infected with *HP* had higher serum LDL levels compared to uninfected animals or those fed a standard diet. These results prompted us to look for a possible link between *HP* infection combined with increased fat intake and arterial or systemic abnormalities in the *Cavia porcellus* model that might promote the development of atherosclerosis.

LDL is a form of cholesterol in the body that is not proatherogenic, however, when it becomes oxidized (ox-LDL), it can stick to the walls of blood vessels, disrupt the function of the cells lining the vessels, and cause inflammation^24^. This happens when ox-LDL interacts with a receptor called LOX-1 on the cells lining the vessels. This interaction triggers a process that produces harmful molecules called reactive oxygen species, which cause damage to cells and can lead to inflammation. One such molecule is 4HNE. At high concentrations, this molecule can cause cell damage or cell death^44^. Our study found that animals infected with *HP* and exposed to high-fat food had ox-LDL deposition in their aortic tissue. This deposition was accompanied by increased levels of 4HNE in the same tissue, as well as higher rates of apoptosis in aortic endothelial cells. This process weakens the vascular endothelial barrier, potentially leading to the progression of atherosclerosis by allowing lipoprotein particles to accumulate inside the vasculature. Ultimately, this can cause even stronger lipid oxidation and deposition, as shown in previous research^45^.

Guinea pigs are a reliable model for studying lipid metabolism and the onset of atherosclerosis induced by a high-fat diet. This is because fatty streaks and atherosclerotic plaques can develop over weeks to months, as shown in a study by Bernick et al. in 1962. The elasticity of the aortic wall in guinea pigs, as in humans, depends on its architecture, which in turn affects systolic pressure^46^. Guinea pigs and other rodent models are becoming increasingly popular for studying the development of arterial stiffness and foam cell formation that occur in atherosclerosis^47–49^. Our data shown in Fig. 6 suggest, that even without a high-fat diet, soluble bacterial components of *HP* can cause the increase of arterial stiffness. We have previously demonstrated that this could potentially be attributed to the deposition of immune complexes^50^. Also, the studies conducted by Laurilla et al.^51^, Satoh et al.^52^ and Vahdat et al.^53^ suggest a significant association between *HP* infection and LDL hypercholesterolemia, HDL hypocholesterolemia, and elevated C reactive protein (CRP) levels. These studies also indicate that chronic infection may have an impact on lipid metabolism, which can increase the risk of CVD. According to Abkas et al. 2010, individuals infected with *HP* have lower serum paraoxonase-1 activity, which is a major anti-atherosclerotic component of HDL, and higher carotid intima-media thickness, which is one of the surrogate markers of atherosclerosis^18^.

The metabolic syndrome can result in cardiovascular disorders, as well as liver disorders known as MASLD. This syndrome is characterized by the buildup of triglycerides in liver cells and the resulting damage, which is similar to that seen in cases of alcohol abuse. However, it is important to note that most patients with the hepatic manifestation of CVD do not abuse alcohol. There are two variants of the disease—simple metabolic dysfunction-associated steatotic liver (MASL) and metabolic dysfunction-related steatohepatitis (MASH). The former is characterized by the accumulation of fat in the liver cells, while the latter is characterized by ballooning and inflammation of liver cells. If left untreated, MASH can lead to liver fibrosis and eventually cirrhosis^54–56^. There is a suggestion that hepatic steatosis can be a useful marker for evaluating early atherosclerotic lesions or vice versa. This is because both diseases share numerous risk factors^57^.

There is some evidence suggesting that *HP* may contribute to the progression of MASLD^58–60^. Pirouz et al.^59^ demonstrated the presence of *HP* DNA in the liver tissue of patients with MASLD. Additionally, Polyzos et al.^60^ detected higher levels of anti-*HP* IgG in patients with MASLD than in those without the condition, indicating a potential association between *HP* and MASLD.

Our study revealed a strong and significant correlation between a high-fat diet, *HP* infection, and the occurrence of hepatic steatosis in *Cavia porcellus*. This condition can be considered as the development of metabolic associated fatty liver disease (MAFLD). In addition, we observed balloon-shaped hepatocytes in liver sections of *HP*/E animals, which may suggest a slow development of non-alcoholic steatohepatitis (NASH). However, we cannot confirm this definitively due to the lack of evidence of lobular inflammation.

Our findings are in agreement with He et al.'s^43^ data. The authors conducted a study on mice and demonstrated that the combination of a high-fat diet and *HP* infection can impact the progression of steatohepatitis. They noted that either infection alone or exposure to a high-fat diet alone did not have a significant impact on hepatic steatosis and the development of MASLD^43^.

ICAM-1 is a glycoprotein that is present on the surface of cells and acts as an adhesion receptor. Its primary function is to regulate the recruitment of leukocytes from the bloodstream to areas of inflammation in the endothelial cells. However, adhesion molecules, such as ICAM-1, are also essential for maintaining endothelial integrity and promoting tissue repair. They do so by facilitating endothelial cell migration, which leads to wound closure and neovascularization^61^. In 2004, Kevil et al. conducted a study on ICAM-1 and its role in regulating endothelial cell motility, which is crucial for the regeneration of endothelial tissue. The study found that ICAM-1-deficient cells exhibited impaired total endothelial cell movement and directional migration. This was associated with reduced phosphorylation of AktThr308 and endothelial nitric oxide synthase Ser1177, as well as lower nitric oxide (NO) bioavailability, which contributed to the motility defect^62^. Studies have demonstrated that when the distribution of ICAM-1 in the endothelium is impaired, it can lead to increased vascular leakage during leukocyte transmigration. This can have potential local and systemic consequences^63^. In addition, when there is a loss of endothelial ICAM-1, wound repair can be impeded due to reduced infiltration of neutrophils and macrophages into the injured area^64^.

In this study, we investigated the impact of high-fat uptake and/or *HP* infection on ICAM-1 expression in arteries, given its crucial role in inflammation. Our findings suggest that chronic *HP* infection alone in *Cavia porcellus* can result in decreased ICAM-1 expression in arteries. We have determined that the observed reduction in ICAM-1 expression in arteries is unlikely due to an endothelial ICAM-1 shedding effect. This is because the levels of systemic sICAM-1, as measured in serum samples, were not increased in animals that were treated with normal feed and inoculated with *HP*, as compared to uninfected animals that received normal feed. It was also noticed that animals that were fed fat-enriched food and exposed to *HP* exhibited a trend towards reduced ICAM-1 expression in arteries when compared to uninfected animals that received only fat-enriched chow.

One limitation of this study is that it cannot provide further explanation for a potential mechanism driven by *HP* that leads to reduced deposition of endothelial ICAM-1. *HP* has various surface components that bind sialic acid or heparan sulphate^65,66^. These components are a part of the endothelial cell glycocalyx, which protects the vascular endothelium and prevents the progression of atherosclerosis-related disorders^67^. There is a possibility that soluble *HP* surface components with such specificity may play a role, but additional studies are required to test this hypothesis.

Our study concludes that the combination of two risk factors: infectious (*HP* infection) and dietary (high-fat diet) may lead to the development of metabolic syndrome, which can cause hepatic steatosis and potentially escalate to metabolic dysfunction-associated steatohepatitis. These findings suggest that *HP* eradication in *HP*-positive individuals could help in the treatment of CVD patients by reducing the risk of atherosclerosis progression by controlling the cascade of events leading to the development and progression of metabolic syndrome. Moreover, *HP* eradication could potentially enable the restoration of ICAM-1-dependent mechanisms of endothelial regeneration in light of *HP*-related increases in oxidative stress and apoptosis.

## Materials and methods

### Animal study

All studies involving animals were approved by the Local Ethics Committee (LKE9) for Animal Experiments of the Medical University of Lodz, Poland, which was established by the Ministry of Science and Higher Education in Poland (58/ŁB45/2016; 44/LB105/2018). Ethics Statement: in vivo experiments were developed according to the EU directive (Directive 2010/63/EU of the European Parliament and of the Council of 22 September 2010 on the protection of animals used for scientific purposes (Dz.U. L 276 z 20.10.2010, s. 33–79)). Adult Himalayan *Cavia porcellus* (350–600 g), free of pathogens, were bred in the Animal House at the Faculty of Biology and Environmental Protection, University of Lodz (Lodz, Poland), kept in cages with free access to drinking water and fed with normal chow or experimental diet (high-fat food). Normal chow for animals contained: proteins (24%), fat (3%), ash (8%), and fiber (10%), together with some nutritional additives, such as Vitamin A, Vitamin D3, Vitamin E, copper, calcium, sodium, phosphorus, threonine, methionine with cysteine and lysine (Agropol, Motycz, Poland). High-fat food contained: fat (19.5%), sucrose (15%), and cholesterol (0.35%) (Vivari, Warszawa, Poland). Animals were inoculated *per os* with *Helicobacter pylori* (*HP),* reference strain CCUG 17874 (Culture Collection University of Gothenburg, Sweden), positive for vacuolating cytotoxin (VacA) and cytotoxin associated gene A (CagA) antigen, which was cultured under microaerophilic conditions or with *Brucella* broth with addition of 10% fetal bovine serum (FBS) (control animals), as previously described^14,68^. Animals were randomly divided into 4 groups (three to five animals per group): C/N (control uninfected/normal diet); C/E (control uninfected/experimental diet); *HP*/N (*HP* infected/normal diet) and *HP*/E (*HP* infected/experimental diet). The feeding of animals intended for *HP* inoculation was discontinued 24 h before the beginning of the experimental procedure, with constant access of animals to drinking water. The control animals – uninfected received 1 mL of 0.2 N NaHCO_3_ by oral route (without anesthesia), and then, after 5 min, 1 mL of *Brucella* broth with FBS (without anesthesia). The procedure was repeated three times at two-day intervals. Animals infected with *HP* received 1 mL of 0.2 N NaHCO_3_ orally (without anesthesia), and then after 5 min, 1 mL of the *HP* suspension 1 × 10^10^ CFU/mL (without anesthesia). The inoculation procedure was repeated 3 times at two-day intervals. Animals were monitored daily (body weight, water, and food intake, behavioral symptoms, skin and fur condition, diarrhea), then on days 60 and 120 were euthanized with an overdose of sodium barbiturate (Morbital, Biowet, Puławy, Poland). Blood samples were proceeded to obtain serum, whereas spleen, liver, and aortal tissue were preserved to perform histopathology. *HP* infection in the animals was confirmed by the presence of Helicobacter-like organisms (HLO) in gastric tissue specimens stained with Mayer hematoxylin and eosin as well as assessment of accompanied local inflammation and the production of anti-*HP* antibodies as routinely done in our laboratory^14,68^. The amount of HLO was graded as: (+++), (++), (+), where + means mild level of colonization/bacteria not detected in every gastric crypt; ++ means moderate level of colonization/bacteria detected in the majority of crypts present; +++ means severe level of colonization/bacteria present in all gastric crypts^68^. The inflammatory reaction was evaluated according to the Sydney system criteria used for histological examination of gastric tissue samples. Fresh tissue specimens were evaluated for urease activity and the isolates were also assessed for the activity of catalase and oxidase. In serum samples the level of anti-*HP* antibodies was assessed by the laboratory enzyme-linked immunosorbent assay (ELISA) using the complex of *HP* antigens called a glycine acid extract (GE)^39^.

Currently in guinea pigs under the study, we also determined the systemic activation of the peripheral immune system, 7 or 28 days from inoculation, by evaluation of the size of spleen germinal centers, which may correlate with activation of innate immune cells, upregulation of inflammation and development of the humoral and cellular adaptive immune response. For this purpose, the thin layer cross-sections of paraffin-embedded aortic tissue after deparaffinization were stained with hematoxylin and eosin or immunohistochemically to visualize surface molecule B220/CD45R, which is a marker characteristic of differentiating B lymphocytes. To assess the presence of B220 in spleen sections, the tissue sections were incubated overnight with primary anti-B220/CD45R antibodies conjugated with fluorescein isothiocyanate (FITC) (dilution 1:500; Thermo Fisher Scientific, Waltham, MA, USA), and then cell nuclei were stained with 4′,6-diamidino-2-phenylindole (DAPI, Sigma-Aldrich, St. Louis, Michigan, USA) for 15 min. After staining spleen sections were imaged using a confocal microscope (Leica SP8, Wetzlar, Germany).

### LDL and soluble ICAM-1 concentration in animal serum samples

The concentration of LDL was measured in animal serum samples with commercial HDL and LDL/VLDL Quantitation Kit according to the manufacturer's protocol (detection range: 2–10 µg/ml; Sigma-Aldrich, St. Louis, Michigan, USA). The concentration of soluble ICAM-1 (sICAM-1) was measured in animal serum samples using commercial Guinea pig sICAM-1/CD54 ELISA Kit assay as recommended by the manufacturer (detection range: 0.313–20 ng/ml; sensitivity min: 0.188 ng/ml, max: 20 ng/ml; MyBioSource, San Diego, California, USA).

### Immunofluorescence for detection of deposition of oxidized LDL, lipid peroxidation, apoptosis, and ICAM-1 exposure in aortic tissue

The thin layer cross-sections of paraffin-embedded aortic tissue were deparaffinized, then incubated with 3% bovine serum albumin (BSA, Sigma-Aldrich, St. Louis, Michigan, USA) in phosphate-buffered saline (PBS)—BSA/PBS, for 30 min at room temperature, to block unspecific binding. To detect deposition of oxidized LDL on aortic tissue, sections were incubated with primary mouse antibodies anti-oxidized LDL (dilution 1:500, Santa Cruz Biotechnology, Dallas, Texas, USA), followed by incubation with secondary fluorescein isothiocyanate (FITC) conjugated goat anti-mouse IgG antibodies (dilution 1:1000, Thermo Fisher Scientific, Waltham, MA, USA). Lipid peroxidation in aortic tissue was assessed by the detection of 4-hydroxy-2-nonenal (4HNE). For this purpose, sections were incubated with FITC conjugated primary mouse anti-4-HNE antibodies, diluted 1:500, and then with Alexa Fluor 594 conjugated mouse anti-CD34 antibodies diluted 1:500 (marker of endothelial cells, Santa Cruz Biotechnology, Dallas, Texas, USA). To visualize cell nuclei sections were incubated with DAPI (Sigma-Aldrich, St. Louis, Michigan, USA), as previously described^69^. Stained tissue sections were imaged using a fluorescent microscope (Zeiss, Axio Scope, A1, Jena, Germany) at appropriate wavelengths: FITC (excitation 495 nm, emission 519 nm), Alexa Fluor 594 (excitation 590 nm, emission 618 nm), and DAPI (excitation 358 nm, emission 461 nm). To detect cells undergoing apoptosis, aortic tissue sections were stained with commercially available enzyme terminal deoxynucleotide transferase dUTP nick labelling (TUNEL) assay (Cat. Number C10617; Thermo Fisher Scientific, Waltham, MA, USA), according to the manufacturer's manual. Stained sections were visualized in a confocal microscope (Leica SP8, Wetzlar, Germany). Fluorescence intensity was measured using ImageJ v1.54 g software (https://imagej.net/ij/index.html). To show the endothelial expression of intracellular adhesion molecule -1 (ICAM-1) sections were stained with primary rabbit antibodies anti-ICAM-1(dilution 1:500, Biorbyt, Cambridge, UK), followed by staining with secondary goat anti-rabbit IgG antibodies conjugated with Alexa Fluor 594 (dilution 1: 2000, Thermo Fisher Scientific, Waltham, MA, USA), and with primary mouse anti-CD34 antibodies (dilution 1:500; Santa Cruz Biotechnology, Dallas, Texas, USA), then with secondary goat anti-mouse IgG antibodies (dilution 1:1000, Thermo Fisher Scientific, Waltham, MA, USA). Cell nuclei were stained with DAPI (Sigma-Aldrich, St. Louis, Michigan, USA). Stained tissues were visualized using a confocal microscope (Leica SP8, Wetzlar, Germany) at appropriate wavelengths: FITC (excitation 495 nm, emission 519 nm), Alexa Fluor 594 (excitation 590 nm, emission 618 nm) and DAPI (excitation 358 nm, emission 461 nm).

### Measurement of artery stiffness

In vivo*,* vascular stiffness was determined in all animals included in the study by photoplethysmography (Indus Instruments, Houston, TX, USA) at the location of the right hind limb artery (after 120 days of the experiment), as previously described in Krupa et al.^14^. Briefly, animals were laid supine on a temperature-controlled plate (THM100, Indus Instruments) and maintained under anesthesia (1% isoflurane) during the procedure. Once the heart rate was stabilized, the amplitudes were measured. The test lasted at least 15–20 s, and at least 20 waves were recorded during this time, but only representative amplitude values were selected to calculate average amplitude (AU) values. Based on the average amplitude values, a score was developed that ranged from 1 to 3; where 1—included amplitude values lower than 1.5 AU, 2—amplitude values between 1.5—2 AU, and 3—amplitude values between 2 and 3 AU.

## Liver histopathology

The liver was sliced with a cryotome (Leica CM1950 Biosystems, Wetzlar, Germany), stained with hematoxylin and eosin, and subjected to histopathological analysis by certified histopathologist (Cytopath, Lodz, Poland). The degree of liver steatosis was evaluated according to a scoring system developed by Kleiner et al.^36^. and was based on five parameters: steatosis grade and location of steatosis/presence of fat droplets, fibrosis, lobular inflammation, and presence of ballooned cells. Additionally, the histopathological evaluation was expanded with the size of steatosis droplets within the organ, of which small-droplet or large-droplet steatosis was distinguished. The scores ranged from 0 to 3, except for the fibrosis category for which they ranged from 0 to 4. The parameters for the hepatic manifestation of the metabolic syndrome, as well as the rules for their evaluation, are shown in Table 1. Each category was evaluated and scored separately.

**Table 1:** Parameters of hepatic manifestation of metabolic syndrome.

Category	Score	Extent
Steatosis grade	0	< 5%
	1	5–33%
	2	33–66%
	3	> 66%
Steatosis localization	0	Zone 3 *
	1	Zone 1 **
	2	Azonal
	3	Panacinar
Hepatocyte ballooning	0	None
	1	Few balloon cells
	2	Many balloon cells
Fibrosis	0	None
	1	Perisinusoidal or periportal
	2	Perisinusoidal and portal/periportal
	3	Bridging fibrosis
	4	Cirrhosis
Lobular inflammation	0	No foci
	1	< 2 foci/200x
	2	2–4 foci/200x
	3	> 4 foci/200x

*Zone 3—hepatocytes encircled portal tracts.

**Zone 1—hepatocytes located around the central veins.

### Scanning electron microscopy (SEM) of liver tissue sections

SEM analysis was performed as described in Rajathi et al. 2022 with minor modifications^70^. Briefly, guinea pig liver tissues were cut into 0.5–1 mm thick slices and then fixed in 2% glutaraldehyde in 0.1 M cacodylate buffer for 1 h, then washed and treated with 1% osmium tetroxide for 1 h. The tissues were then dehydrated using an ethanol series (10%, 30%, 50%, 70%, 80%, 96%, 99.6%). Dehydrated tissues were dried using a Leica EM CPD300 critical point dryer (Leica Microsystems GmbH, Vienna, Austria). Liver sections were mounted using carbon adhesive tabs and imaged using a Phenom ProX scanning electron microscope (Thermo Fisher Scientific Inc., Waltham, USA).

## Statistical analysis

All graphs were performed using GraphPad Prism 10.1.2(324) software (https://www.graphpad.com/features) and values were expressed as the mean values with standard deviation apart from artery stiffness measurement presented as the mean values with standard error of the mean. The Shapiro–Wilk test was used to assess the Gaussian distribution. The Brown-Forsythe test was used to verify the equality of group variances. For two- and three- way ANOVA analysis, Spearman`s test for heteroscedasticity was used. Data of oxLDL deposition were log transformed before analysis to attain heteroscedasticity of residuals. The individual tests are described below the figures. In the case of all statistical analysis performed, alpha was set to 0.05.

## Data Availability

All data generated or analyzed during this study are included in this published article.
